# Mucin levels in glands of the colonic mucosa of rats with diversion colitis subjected to enemas containing sucralfate and n-acetylcysteine alone or in combination

**DOI:** 10.1590/acb384023

**Published:** 2023-10-13

**Authors:** Verena Palmeiras Brasil, Rayama Moreira Siqueira, Fabio Guilherme Campos, Mateus Magami Yoshitani, Geovanna Pacciulli Pereira, Roberta Laís dos Santos Mendonça, Danilo Toshio Kanno, José Aires Pereira, Carlos Augusto Real Martinez

**Affiliations:** 1 Universidade Estadual de Campinas – Postgraduate Program in Surgical Sciences – Campinas (São Paulo) – Brazil.; 2 Universidade de São Paulo – Department of Gastroenterology – Faculty of Medicine – São Paulo (São Paulo) – Brazil.; 3 Universidade São Francisco – Faculty of Medicine – Medical School – Bragança Paulista (São Paulo) – Brazil.

**Keywords:** Colitis, Sucralfate, Acetylcysteine, Mucins, Image Processing, Computer-Assisted, Models, Animal

## Abstract

**Purpose:**

To evaluate the tissue content of neutral and acidic mucins, sulfomucins and sialomucins in colonic glands devoid of intestinal transit after enemas containing sucralfate and n-acetylcysteine alone or in combination.

**Methods:**

Sixty-four rats underwent intestinal transit bypass. A colonic segment was collected to compose the white group (without intervention). After derivation, the animals were divided into two groups according to whether enemas were performed daily for two or four weeks. Each group was subdivided into four subgroups according to the substance used: control group: saline 0.9%; sucralfate group (SCF): SCF 2 g/kg/day; n-acetylcysteine group (NAC): NAC 100 mg/kg/day; and SCF+NAC group: SCF 2 g/kg/day + NAC 100 mg/kg/day.Neutral and acidic mucins were stained by periodic acid-Schiff and alcian-blue techniques, respectively. The distinction between sulfomucins and sialomucin was made by the high alcian-blue iron diamine technique. The content of mucins in the colonic glands was measured by computerized morphometry. The inflammatory score was assessed using a validated scale. The results between the groups were compared by the Mann-Whitney’s test, while the variation according to time by the Kruskal-Wallis’ test (Dunn’s post-test). A significance level of 5% was adopted.

**Results:**

There was reduction in the inflammatory score regardless of the application of isolated or associated substances. Intervention with SCF+NAC increased the content of all mucin subtypes regardless of intervention time.

**Conclusions:**

The application of SCF+NAC reduced the inflammatory process of the colonic mucosa and increased the content of different types of mucins in the colonic glands of segments excluded from fecal transit.

## Introduction

Surgeons frequently perform temporary colostomy surgery in patients with a variety of diseases. However, in many of these patients, due to different situations, intestinal transit is never restored, causing the temporary stoma to become permanent^1^. Permanently living with a stoma greatly impacts the quality of life of these patients^1^. In addition to the limitations due to the presence of the stoma, most of these patients develop a series of metabolic, histological, and molecular changes in the colonic segment excluded from fecal transit called diversion colitis (DC)^2^.

The development of mucosal inflammation in colonic segments excluded from fecal transit was first described in 1974 by Morson and Dawson^3^. This new form of colitis was forgotten for almost a decade, until in 1981 Glotzer et al.^4^ described the disease in detail in 10 patients who did not have previous inflammatory diseases and who developed inflammation in the excluded segments, which was reversible in five patients as a result of fecal transit restoration. Subsequently, a series of clinical and experimental studies showed that the molecular mechanisms that determine the inflammatory process in the mucosa of the colon are related to a deficiency in the main energy sources, short-chain fatty acids (SCFAs), and, more particularly, butyric acid, for the cells that compose the colonic glands^5^,^6^.

Decreases in the regular supply of SCFAs cause epithelial cells to modify their metabolism and start using alternative sources for energy^7^,^8^. This metabolic modification triggers the overproduction of reactive oxygen and nitrogen species (RONS). (RONS), toxic to cells that comprise the colonic epithelium, causing the breakdown of the colonic epithelial barrier.

The colonic epithelium is one of the most functional barriers in the human body. Formed by only one layer of juxtaposed cells and covered by a specialized mucus layer, it forms the first line of protection against the infiltration of pathogens present in the intestinal microbiota. The mucus layer that covers the colonic epithelium is continuously produced by goblet cells as long as the energy supply for protein synthesis is maintained. However, in situations of energy supply deficiency, such as DC, the production of its most important constituent glycoprotein decreases considerably^9^,^10^.

The mucus that covers the intestinal epithelium is composed of glycoproteins called mucins^11^. The mucin molecule is composed of a carbohydrate and a protein moiety^12^. The mucins are subdivided into neutral or acidic mucins according to the presence of glycogen or sialic acid in their glucose fraction, respectively. While neutral mucins are predominantly found in the cranial portions of the digestive tract, acidic mucins are majorly found in the distal colon. Acidic mucins are subdivided into sialomucins and sulfomucins, according to the presence of sialic acid or sulfate groups. Sialomucins are characterized according to the proportion of N-acetyl or O-acetyl molecules derived from sialic acid^13^,^14^. The lack of SCFA supply, in addition to the considerable reduction in the ability of goblet cells to synthesize mucins, increases the formation of RONS. These free radicals destroy the mucus layer that covers the epithelial barrier and induce goblet cells apoptosis in the intestinal glands, triggering and exacerbating the inflammatory process that characterizes DC^15^,^16^.

Studies have evaluated the use of different substances with antioxidant action for the treatment of DC using experimental DC models. Among them, the intrarectal application of enemas containing n-acetylcysteine (NAC) or sucralfate (SCF) showed promise^17^,^18^. Both substances reduced the levels of oxidative stress in the mucosa of the excluded colon, suppressed the inflammatory process, and promoted epithelial healing^17^-^20^. Despite having a remarkable antioxidant effect, due to its low ability to adhere to the colonic mucosa, NAC requires successive enema applications, which made its use impractical^18^,^19^. In contrast, SCF, in addition to stimulating mucin production by goblet cells, can effectively adhere to the inflamed colonic mucosa^17^,^21^,^22^. SCF adheres firmly to the mucosa, forming a gelatinous barrier over the inflamed surface. It was demonstrated that the application of SCF increases the production of mucins in the colonic epithelium excluded from fecal transit, decreases the inflammatory process, and promotes mucosal healing^21^,^22^. Its high adhesive capacity presents the additional advantage of reducing the need for several applications per day^22^,^23^. However, when compared to NAC, SCF has lower antioxidant action^20^,^24^,^25^.

In view of the evidence showing that both substances have complementary properties, the present study was designed to evaluate whether the application of enemas containing both substances is able to reduce the inflammatory process and increase the production of the different mucin subtypes that coat the excluded colonic epithelium.

## Methods

### Ethics statement

This study followed the recommendations of Federal Law No. 11,794 and the guidelines of the Brazilian College of Animal Experimentation. The research project was submitted to the Ethics Committee on the Use of Animals in Research of the Universidade São Francisco, with the approval number 001.226.2014.

### Diversion colitis induction and experimental groups

During December 2019 to January 2020, 64 male Wistar rats, weighing between 300 and 350 g and aged between four and five months old, were kept in individual cages in an air-conditioned environment with controlled temperature, light, humidity and noise conditions for seven days to acclimate to the central animal facility of the Universidade São Francisco. During the experiment, all animals were fed a diet suitable for rodents (Nuvilab CR1, Nuvital Nutrientes S/A, Colombo, PR, Brazil) and weighed weekly. The mice were fasted for 12 hours on the day before intestinal transit bypass surgery, but they still had access to water.

Anesthesia was performed using 2% xylazine hydrochloride (Anasedan Agribrands do Brasil Ltda., Brazil) + ketamine hydrochloride (DopalenAgribrands do Brasil Ltda., Brazil), which was intraperitoneally administered at a dose of 0.1 mL/100 g. After shaving the entire anterior region of the abdomen, the abdominal cavity was accessed using a 4-cm longitudinal median infraumbilical incision. After opening the abdominal cavity, Peyer’s patch, a lymphoid structure located in the anterior wall of the large intestine, was identified, which was used to repair the colon section. After ligation and sectioning of the juxtacolic marginal artery, the colon was sectioned 8 cm above the cranial end of the Peyer’s patch. At this site, a 2-cm segment of the large intestine was removed to form the white Group (colic segment with preserved fecal transit). After collection of the control colon segment, the proximal colon on the left flank of the abdominal wall was subjected to terminal colostomy. The distal segment of the sectioned large intestine was catheterized with a 10F polyvinyl probe fixed with a loose ligature and irrigated with 40 mL of 0.9% saline solution at 37°C until the effluent fluid from the animal’s anus no longer contained fecal residues. At the end of this stage, the distal colon was redirected into the left iliac fossa as a distal mucous fistula. The abdominal wall was then closed with two types of suture. After recovery from anesthesia, the animals were allowed to drink water, and after 6 hours, they were provided with chow.

**Figure 1:**
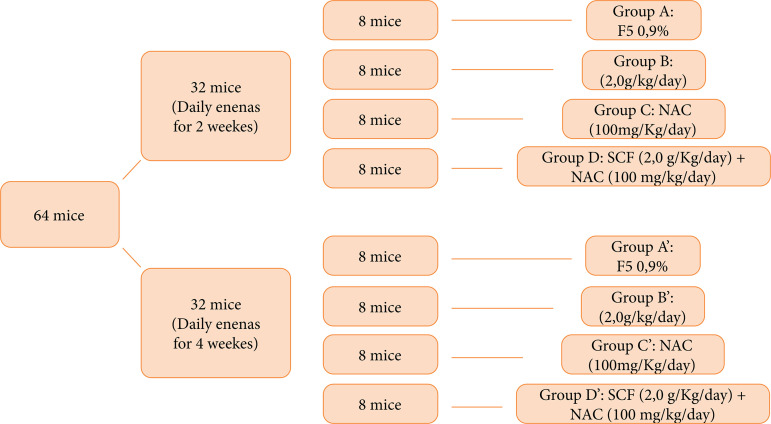
Groups and subgroups according to time intervention and substance used.

Source: Elaborated by Carlos Augusto Real Martinez. SCF: sucralfate; NAC: n-acetylcysteine.

The 64 animals were divided into two experimental groups with 32 animals each according to the daily enemas with the proposed substances for two or four weeks. Each of these two groups, in turn, was divided into four subgroups containing eight animals according to the intervention substance used. Thus, 32 animals were subjected to daily application of enemas containing 0.9% physiological saline (FS); SCF 2 g/kg/day; NAC 100 mg/kg/day; and SCF 2 g/kg/day + NAC 100 mg/kg/dayfor two weeks, and the remaining 32 mice were treated with the same substances at the same dosage for four weeks. The subgroups and interventions performed are described in Fig. 1.

### Material collection and euthanasia

On the dates scheduled for euthanasia (two or four weeks), the animals were again anesthetized with the same technique described before. The samples removed from the colon without fecal transit submitted to the interventions were opened longitudinally by the antimesenteric border. Fragments of the excluded colon, measuring 2 cm in length, were removed, and fixed with the mucosal surface facing up on a cork fragment. Then, they were placed in individual plastic bottles, previously marked with the animal number and the experimental group to which they belonged, containing 10% buffered formaldehyde solution for fixation.

Euthanasia of the animals followed the guidelines of the Brazilian College of Experimentation with Animals and the Federal Council of Veterinary Medicine, and the animals were anesthetized by intracardiac administration with a lethal dose of sodium thiopental (120 mg/kg). The death of the animals was confirmed by the absence of vital signs such as heartbeat and loss of the corneal-palpebral reflex.

### Histological technique

The colon segments were placed in bottles containing 10% buffered formaldehyde solution (Sigma, St. Louis, MO, United States of America) and kept for 72 hours. When the fixation process was complete, they were removed, washed in distilled water, and dehydrated with ethanol and xylene solutions. Then, they were embedded in paraffin. After the paraffin blocks were ready, they were sectioned longitudinally with a microtome to a thickness of 5 μm for staining.

The hematoxylin-eosin (HE) staining technique was used to identify the presence of colitis and to grade the inflammatory score. The collagen levels in the tissues were evaluated using Masson’s trichrome staining. For the identification of neutral mucins, the periodic acid-Schiff (PAS) staining techniqu e was used. The acidic mucins were stained using the Alcian blue technique, and the high-Alcian blue iron diamine (HIDAB) technique was used to differentiate the subtypes of acidic mucins, sulfomucins, and sialomucins, as previously described^14^,^16^.

### Analysis of histological slides

For the histological evaluation of the degree of inflammation, the slides were evaluated by a pathologist experienced in colorectal diseases who was blinded to the origin of the material and the objectives of the study. The slides were always observed under a common optical microscope with a final magnification of 200x.

To standardize the area of interest, the slides were always analyzed in the same location 5 cm above the anal canal. The variables used to assess the degree of tissue inflammation were loss of epithelial surface (epithelial ulcerations), abscesses in colic crypts, intensity of inflammatory infiltrate, and presence of epithelial fibrosis, the first three of which were stratified into crosses, according to the following categories:

- 0: no change;
- +: light;
- ++: moderate;
- +++: intense.

The intensity of tissue fibrosis was evaluated by the total tissue collagen level identified by Masson’s trichrome stain and stratified according to the percentage found in the studied histological field, according to the follow categories:

- 0: no fibrosis;
- 1: fibrosis ≥ 1 and ≤ 5%;
- 2: fibrosis > 5 and ≤ 10%;
- 3: fibrosis > 10%.

The final value for each animal was the mean after the quantification of three different histological fields for all the variables analyzed.

### Computer-assisted image analysis

The tissue levels of neutral mucins, acidic mucins, sulfomucins and sialomucins were measured by computer-assisted image analysis (computed morphometry). For each animal, the tissue protein level was quantified in three different histological fields where there were at least three intact and contiguous colonic glands. The selected image, after proper focusing, was captured by a video camera (DS-Fi, Nikon Instruments Inc., Japan) coupled to an optical microscope (Elipse-50i, Nikon Instruments Inc., Japan). The captured image was then processed and analyzed using NIS-Elements software (Nikon Instruments, Japan) installed on a computer with suitable image processing capacity.

In the program, magenta staining represented the tissue expression of neutral mucins, blue staining represented the tissue expression of acidic mucins, and sialomucins (identified by HIDAB staining) and brown staining, representing the tissue expression of sulfomucins (also identified by HIDAB staining), was replaced by white, while the remaining field of view was captured in black and white, forming a binary image. The image analysis program automatically calculated how many white pixels were present in the black total histological field. The values found for the tissue levels of the studied glycoproteins were always expressed as the percentage of pixels (protein studied) per field analyzed (%/field). The final value for the animals belonging to the control and experimental groups was always represented by the mean value, with the respective standard error.

### Statistical methods

The data are presented as the mean ± standard error of the mean. The T test was used to evaluate the changes in body weight. The Mann–Whitney’s test was used to compare the inflammatory grade score and the tissue levels of neutral mucins, acidic mucins, sulfomucins and sialomucins in a paired manner. For all tests, a significance level of 5% (p £ 0.05) was used. The significant values when comparing the results of the intervention with the different substances in the same period (two or four weeks) were marked with an * when the significance level was lower than 5% or with ** when the significance level was less than 1%. The Kruskal–Wallis’ test was used to analyze the variance in the results found in each experimental group with respect to the intervention time (two or four weeks). Dunn’s post-test was used to verify the significance between the groups in the analysis of variance. The statistical evaluation of intervention time in each experimental group was also performed in a paired manner. The significant values when comparing the intervention periods (two or four weeks) of animals treated with different substances were marked with † when the significance level was lower than 5% or with †† when the significance level was less than 1%.

## Results

The graphs show the paired comparison between the intervention groups and the control group within the same intervention time: two or four weeks. The tables following each graph show the variation in the results found with the use of enemas containing the same intervention solution (blank, control, SCF, NAC, SCF+NAC) in relation to the intervention time.

**Figure 2:**
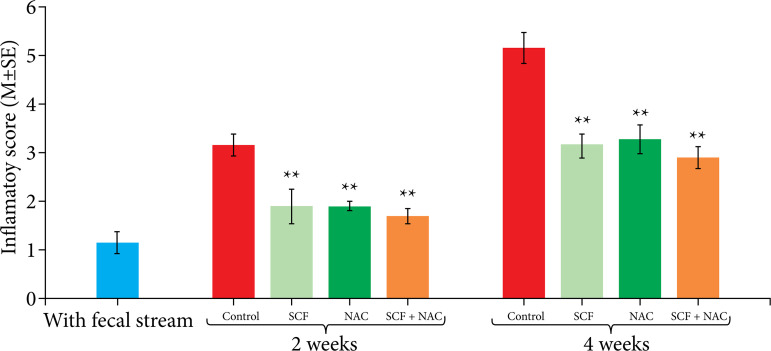
Comparison of the inflammatory score in the control group and the saline, SCF, NAC and SCF+NAC treatment groups at two or four weeks of intervention.

Source: Elaborated by Carlos Augusto Real Martinez. **p < 0.01 (Mann–Whitney’s test); SCF: sucralfate; NAC: n-acetylcysteine.

Figure 2 shows the mean, with the respective standard error of the inflammatory score values of the different experimental subgroups at the two intervention times considered (two or four weeks). Animals that received enemas containing SCF, NAC and SCF+NAC exhibited a reduced inflammatory score compared to that of animals that received enemas containing 0.9% saline, regardless of the intervention time.

Table 1 shows the variation in the inflammatory score (IS) comparing the same subgroup according to intervention time. Although the inflammatory score increased after four weeks of intervention, it was found to be lower in animals treated with SCF, NAC and SCF+NAC than in animals that received enemas containing 0.9% saline.

**Table 1:** Mean inflammatory score variation, with respective standard error, in relation to intervention time (two or four weeks).

Experimental group	I.S. (M±S.E.)	
	2 weeks	4 weeks
White group	1.16±0.16	-
Saline	3.12±0.22	5.11±0.26†
SCF	1.87±0.35	3.11±0.26†
NAC	1.88±0.11	3.22±0.27†
SCF+NAC	1.66±0.16	2.88±0.11†

I.S.: inflammatory score; M: mean; S.E.: standard error;

† p<0.05 Kruskal-Wallis’ test and Dunn’s post-test; SCF: sucralfate; NAC: n-acetylcysteine.

Source: Elaborated by Carlos Augusto Real Martinez.

**Figure 3:**
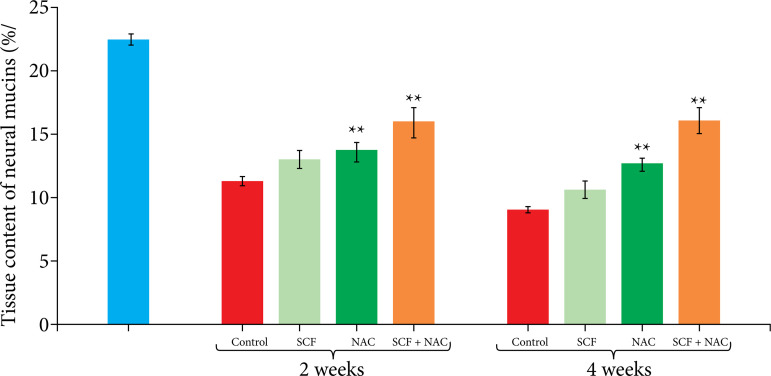
Comparison of the tissue neutral mucin level as percentage per field (%/field) in the control group and in the animals subjected to intervention with saline, SCF, NAC and SCF+NAC for two or four weeks.

Source: Elaborated by Carlos Augusto Real Martinez. **p < 0.01 (Mann–Whitney’s test); SCF: sucralfate; NAC: n-acetylcysteine.

Figure 3 shows, on average, with the respective standard error, the tissue levels of neutral mucins, in percentage per histological field (%/field), between the different experimental subgroups at the two tested intervention times (two or four weeks). The neutral mucin level increased in animals that received enemas containing NAC or SCF+NAC, but it did not change in animals treated with SCF alone both at two and at four weeks of intervention. In animals treated with the two drugs, the neutral mucin level was higher.

Table 2 shows the variation in neutral mucin levels within the same subgroup according to intervention time. There was no difference in the neutral mucin level between the intervention time.

**Table 2:** Variation in mean neutral mucin level, with respective standard error, in relation to intervention time (two or four weeks).

Experimental group	Neutral mucin (M±S.E.)	
	2 weeks	4 weeks
White group	22.43±0.43	-
Saline	11.24±0.33	9.04±0.26†
SCF	12.94±0.35	10.55±0.27
NAC	13.59±0.78	12.60±0.55
SCF+NAC	15.87±1.20	16.06±1.01

M: mean; S.E.: standard error;

† p<0.05 Kruskal-Wallis’ test and Dunn’s post-test; SCF: sucralfate; NAC: n-acetylcysteine. Source: Elaborated by Carlos Augusto Real Martinez.

**Figure 4:**
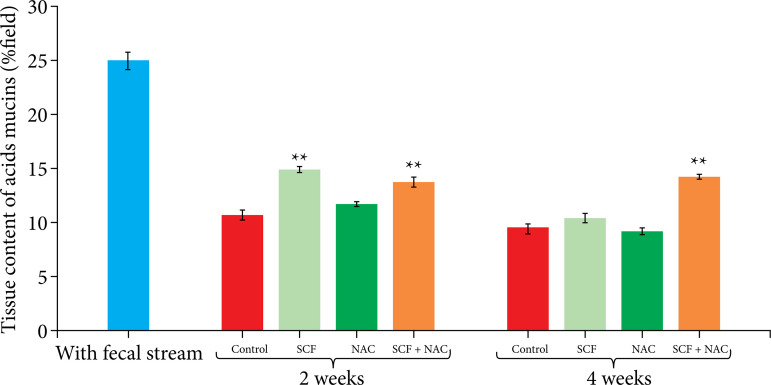
Comparison of the tissue acid mucin level in percentage per field (%/field) in the control group and in the animals subjected to intervention with saline, SCF, NAC and SCF+NAC for two or four weeks.

Source: Elaborated by Carlos Augusto Real Martinez. **p < 0.01 (Mann–Whitney’s test); SCF: sucralfate; NAC: n-acetylcysteine.

Figure 4 shows the average, with the respective standard error, of the total tissue acid mucin level in percentage per histological field (%/field), between the different experimental subgroups at the two intervention times (two or four weeks). Animals treated with SCF alone showed higher acid mucin levels after two weeks of intervention. However, animals treated with SCF+NAC had higher acid mucin levels at both two and four weeks of intervention.

Table 3 shows the variation in acid mucin levels between the same subgroup according to intervention time. There was no variation in the acid mucin level regardless of the intervention solution used as a result of different intervention periods.

**Table 3:** Variation in mean acid mucin level, with respective standard error, in relation to intervention time (two or four weeks).

Experimental group	Acid mucin (M±S.E.)	
	2 weeks	4 weeks
White group	24.96±0.77	-
Saline	10.59±0.49	9.54±0.38
SCF	14.89±0.26†	10.48±0.30
NAC	11.69±0.31†	9.22±0.33
SCF+NAC	13.72±0.53	14.19±0.25

M: mean; S.E.: standard error;

† p<0.05 Kruskal-Wallis’ test and Dunn’s post-test; SCF: sucralfate; NAC: n-acetylcysteine. Source: Elaborated by Carlos Augusto Real Martinez.

**Figure 5:**
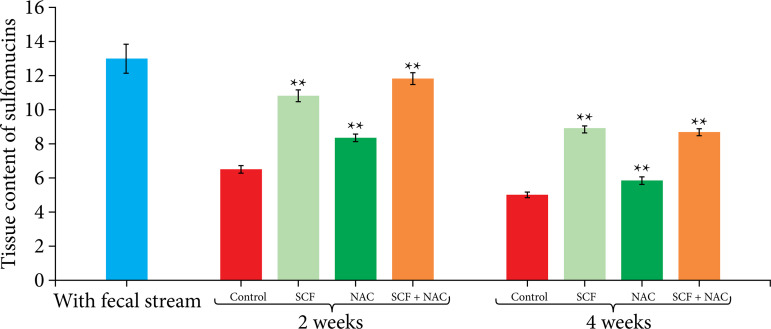
Comparison of the tissue sulfomucin levels as percentage per field (%/field) in the control group and in the animals subjected to intervention with saline SCF, NAC and SCF+NAC for two or four weeks.

Source: Elaborated by Carlos Augusto Real Martinez. **p < 0.01 (Mann–Whitney’s test); SCF: sucralfate; NAC: n-acetylcysteine.

Figure 5 shows the mean, with the respective standard error, of the total tissue level of sulfomucins, as a percentage per histological field (%/field), between the different experimental subgroups at the two tested intervention times (two or four weeks). When compared to animals that received only 0.9% saline, animals that received each substance exhibited significantly increased sulfomucin levels, both at two and four weeks of intervention.

Table 4 shows the variation in sulfomucin levels between the same subgroup according to intervention time. The sulfomucin level was higher in animals treated for two weeks, regardless of the intervention solution used.

**Table 4:** Variation in mean sulfomucin level, with respective standard error, in relation to intervention time (two or four weeks).

Experimental group	Sulfomucin (M±S.E.)	
	2 weeks	4 weeks
White group	12.96±0.86	-
Saline	6.50±0.24†	5.02±0.18
SCF	10.83±0.32†	8.85±0.19
NAC	8.34±0.23†	5.86±0.22
SCF+NAC	11.78±0.32†	8.64±0.25

M: mean; S.E.: standard error;

† p<0.05 Kruskal-Wallis’ test and Dunn’s post-test; SCF: sucralfate; NAC: n-acetylcysteine. Source: Elaborated by Carlos Augusto Real Martinez.

**Figure 6:**
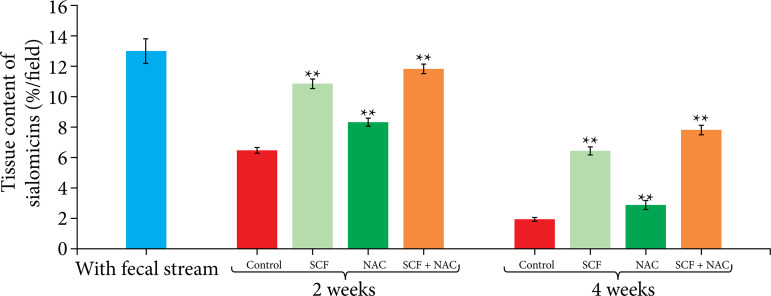
Comparison of tissue sialomucin levels as percentage per field (%/field) in the control group and in animals subjected to intervention with saline, SCF, NAC and SCF+NAC for two or four weeks.

Source: Elaborated by Carlos Augusto Real Martinez. **p < 0.01 (Mann–Whitney’s test); SCF: sucralfate; NAC: n-acetylcysteine.

Figure 6 shows the mean, with the respective standard error, of the values of the total tissue content of sialomucins, in percentage per histological field (%/field), comparing the different experimental subgroups at the two intervention times considered (two or four weeks). Regardless of the drug used, there was an increase in the tissue content of sialomucins when comparing animals treated with SCF, NAC or SCF+NAC with those that received enemas containing only saline. This increase in sialomucin content was maintained both at two weeks and after four weeks of intervention.

Table 5 shows the variation in sialomucin levels between the two intervention times. The sialomucin level was higher in treated animals that received the treatment solutions for two weeks.

**Table 5:** Variation in mean sialomucin level, with respective standard error, in relation to intervention time (two or four weeks).

Experimental group	Sialomucin (M±S.E.)	
	2 weeks	4 weeks
White group	12.96±0.86	-
Saline	6.04±0.24†	1.99±0.13
SCF	10.03±0.32†	6.43±0.28
NAC	8.34±0.23†	2.92±0.17
SCF+NAC	11.78±0.36†	7.82±0.33

M: mean; S.E.: standard error;

† p<0.05 Kruskal-Wallis’ test and Dunn’s post-test; SCF: sucralfate; NAC: n-acetylcysteine. Source: Elaborated by Carlos Augusto Real Martinez.

## Discussion

The mucus layer that covers the colonic epithelium represents the first line of defense against the development of a number of inflammatory bowel diseases. A deeper understanding of the mucus layer and the mechanical barrier of the colonic epithelium has attracted the attention of the scientific community in recent years^26^. With the discovery of new factors that are involved in the relationship between the intestinal microbiota and the intestinal epithelium, the role played by the mucus layer becomes increasingly relevant^26^.

The mucus that covers the intestinal mucosa has a double-layer structure^27^. The first layer is firmly adhered to the mucosal epithelium, while the second layer, superimposed on the first one, can be easily removed from the epithelial surface^28^. This double layer is formed predominantly by mucins, a class of glycoproteins responsible for the protective function^29^,^30^. The mucins found throughout the gastrointestinal tract belong to two main groups according to their molecular glucose fraction: neutral mucins, rich in glycogen, and acidic mucins, rich in sialic acid^11^. Acidic mucins have two main subtypes: sulfomucins, rich in sulfate radicals, and sialomucins, which have a high sialic acid level. Sialomucins are more commonly found in the colon and rectum, while sulfomucins are found in the proximal colon^11^,^13^. When considering the protein fraction of mucins, they are classified into two large groups: those that form a gel when secreted that coats the mucosa (MUC-2, MUC-5AC, MUC-5B, and MUC-6) and those that are present in the membrane (MUC-1, MUC-3, and MUC-4)^31^.

The supply of SCFA, and particularly butyric acid, to mucosal cells is essential for the expression of genes related to the transcription of the mucin protein fraction^32^,^33^. It was demonstrated that there is a decrease in mucin synthesis in the colonic epithelium in all clinical conditions that reduce the supply of SCFAs to the epithelial cells of the colonic mucosa^34^,^35^. Thus, in the colon segment excluded from the intestinal transit, the synthesis of mucins decreases, most likely due to the reduction in both the glucose and protein fractions that comprise the necessary glycoproteins^6^,^32^,^33^,^36^,^37^.

Experimental studies conducted in the 1990s using models of colitis induced by the simple deprivation of SCFA supply in the mucosal cells of the large intestine showed that there are important changes in the tissue distribution and expressional level of mucins^11^,^29^,^31^,^36^,^38^. The synthesis of both mucin glucose and protein fractions was shown to be significantly reduced in goblet cells of the colonic glands when devoid of regular SCFA supply^11^,^13^,^31^. As a result, changes occur in the first line of epithelial defense, making it vulnerable to the action of pathogens present in the colonic lumen.

Keli et al.^36^ were the first authors to study changes in the expression pattern and level of acidic mucins in the large intestine segments excluded from fecal transit. In this pioneering study, the authors evaluated the expression pattern and levels of mucins in rats, comparing segments included and excluded in intestinal transit. They found a reduction in the levels of both subtypes of acidic mucins in the segments excluded from fecal transit, in addition to the disappearance of sialomucins as the inflammatory process worsened in the excluded colon. However, it is important to note that, in this study, the tissue level was subjectively evaluated, and mechanical preparation of the colon excluded from transit was not performed when the authors performed the bypass^36^.

Nonose et al.^11^, aiming to evaluate the lack of a regular supply of SCFAs in mucin synthesis, evaluated the level of the mucin glycidic fractions by comparing colonic segments included and excluded from fecal transit for six, 12 and 18 weeks. The authors carefully washed the colonic segment that was excluded from fecal transit to ensure that fecal residues and, consequently, SCFA would not be present in the excluded colorectal segment. The authors also used a sophisticated computer-assisted image analysis system to measure the levels of the main mucin subtypes. They found that, compared to colonic segments with preserved fecal transit, there was a significant reduction in the tissue levels of neutral and acidic mucins in the colon without SCFA supply^11^. The same group later confirmed these findings, showing that the reduction in the acid mucin levels was mainly due to the reduction in sialomucin synthesis^13^.

Experimental studies suggest that other factors may cause reduction in mucin levels in the colon deprived of the regular supply of SCFAs. SCFA deficiency triggers inflammation in the excluded colonic segments^39^,^40^. The mucosal inflammatory process has been shown to be related to changes in cellular metabolism imposed by the lack of a regular supply of SCFAs as a result of the diversion of fecal transit^8^,^39^,^41^-^48^. This deficiency causes an important change in the mechanisms of β-oxidation to produce the energy required to supply the different phases of the cell cycle^7^,^39^,^46^,^49^,^50^. Thus, the cells of the colonic mucosa modify their mitochondrial mechanisms to obtain energy, forming a large amount of RONS^48^. The overproduction of RONS, together with the deficiencies of the antioxidant systems present in the colonic mucosa, determines tissue oxidative stress^39^,^51^.

A series of clinical and experimental studies have shown that the increase in RONS production due to the lack of SCFA supply is one of the main mechanisms that leads to the breakdown of the different components that compose the functional barrier of the colonic epithelium^16^,^39^,^48^. This dysfunction is one of the most recent explanations for the initial molecular mechanisms that trigger inflammatory bowel diseases known as the theory of induction of colitis by free radicals^48^. Given this evidence, a series of experimental studies showed that the induction of colitis by free radicals could also explain the pathogenesis of DC^11^,^13^,^31^,^52^-^55^.

The possibility that oxidative stress is the mechanism responsible for the development of DC was supported by a series of experimental studies showing that the administration of substances with antioxidant activity, in addition to reducing the levels of tissue oxidative stress, alleviated mucosal inflammation and reestablished the integrity of the different proteins that form the mucus layer of the epithelial barrier^17^-^22^,^25^,^52^,^56^-^59^.

Among the tested substances, NAC was shown to be very effective in reducing tissue oxidative stress and alleviating inflammation in the excluded colon segments^18^. Experimental studies that used the application of enemas containing NAC in segments excluded from fecal transit that developed DC showed that the substance was able to significantly reduce the tissue levels of RONS and reverse the inflammatory changes in the colonic mucosa^18^,^59^. However, as NAC has low adhesion capacity to the inflamed epithelial surface, there was a need for repeated daily applications of the enemas, making its use in clinical practice difficult due to the need to repeat the enemas several times a day^24^. To solve this issue, SCF, which has a high capacity for adhesion to the inflamed mucosal surface, can be administered in combination. Thus, the need for frequently administered enemas could be reduced^24^.

SCF is a substance used for the treatment of different diseases that affect the gastrointestinal tract. Its main mechanism of action is related to its great ability to firmly adhere to the inflamed mucosal surface^18^,^19^,^21^,^22^. SCF is considered a cytoprotective agent and is initially used in the prevention or treatment of peptic ulcers, acute lesions of the gastric mucosa and chronic skin wounds^25^. It also has a remarkable ability to increase prostaglandin E2 production, stimulate mucin production, increase epithelial growth factor synthesis, and induce healing of mucosal lesions, in addition to having antibacterial, anti-inflammatory and, particularly, antioxidant activity^12^,^17^,^25^.

A series of experimental studies showed that the application of enemas containing SCF in colonic segments excluded from intestinal transit that developed DC improved the mucosal inflammatory process, reduced the severity of tissue oxidative stress, stimulated the production of different mucin subtypes, and restored the integrity of the intercellular junction mechanisms^17^,^21^,^22^,^23^,^25^. Despite its great adhesive capacity to the surface of the inflamed colonic mucosa, its antioxidant effect did not have the same potency as that of NAC^18^.

When considering the outstanding antioxidant activity of NAC and the great ability of SCF to adhere to the inflamed colonic mucosa, perhaps the application of the two substances in combination could be more effective than the use of enemas containing the two substances alone^12^,^18^,^21^,^22^,^25^. However, reviewing the literature, to date, no studies evaluating the effects of the application of enemas containing both NAC with SCF on the preservation of mucin levels in the mucosa of segments of the colon excluded from fecal transit with DC have been found. If this formulation can increase, or at least preserve, the level of the different mucin subtypes in the excluded inflamed mucosa, this would be initial evidence of that the combination of the two substances may be beneficial for the treatment or prevention of DC.

The results of the present study showed that the application of daily enemas containing SCF, NAC or SCF+NAC was able to reduce the intensity of the inflammatory score in the colonic mucosa without intestinal transit, regardless of the intervention time. When compared to animals that received enemas containing only saline, the combination of both substances significantly reduced the inflammatory process, although this difference was not greater when compared to the use of each substance alone. These findings corroborate previous studies showing the efficacy of both substances in improving the inflammatory process in the mucosa of the excluded colon^17^,^18^,^21^,^22^.

When analyzing the effects of the enemas on the tissue levels of neutral mucins in animals subjected to intervention with SCF+NAC, it was found that there was a significant increase in neutral mucin levels regardless of the time of intervention. These findings suggest that there was synergism between the two substances. This effect may be related to a greater reduction in tissue oxidative stress in the mucosa of the colonic segments excluded from fecal transit, as previously demonstrated in an DC model similar to the one used in the present study^18^,^20^,^21^,^22^. The greater ability of SCF to adhere to the colonic mucosa is likely to have prolonged the action time of the drugs, particularly NAC, in the colonic mucosa and that this longer action also contributed to a greater removal of RONS and, consequently, led to minimal destruction of the produced mucins. Moreover, when measuring the total acid mucin levels after the enema therapies, it was found that the acid mucin levels in animals subjected to intervention with SCF+NAC showed a significant increase in the colon excluded from fecal transit, regardless of the intervention time.

When analyzing the acid mucin subtypes, it was found that the sulfomucin levels in the segments with preserved fecal transit were higher in the colonic tissue. These findings probably reflect the importance of regular SCFA supply for the synthesis of this mucin subtype. When comparing the sulfomucin levels in animals that received saline, SCF, NAC or SCF associated with NAC, it was found that, regardless of whether the substances were used alone or in combination, there was a significant increase in the levels of sulfomucins regardless of the intervention time. Interestingly, the sulfomucin level decreased over time, regardless of the substance used in the enemas. The higher level of sulfomucins in the first two weeks of intervention may be related to both the reduction in the levels of oxidative stress caused by the substances and to a residual capacity of sulfomucin synthesis by the cells present in the colonic mucosa. Similar findings have been previously described when the SCF intervention was performed for two weeks^22^.

When quantifying the tissue levels of sialomucins, it is important to note that the application of enemas containing SCF+NAC was more effective in maintaining the sialomucin levels when compared to the use of the substances alone. It is possible that these findings are related to the greater antioxidant activity when the two substances are used in combination, as well as the greater permanence of the drugs in contact with the mucosa of the excluded colon.

It should be noted that this study has limitations. This is an experimental study in which the results cannot be always extrapolated to humans. In addition, for ethical reasons, experimental groups were formed with a small number of animals, which increases the chance of type I error in the tissue levels of mucin subtypes in experimental models of DC. To the best of our knowledge, there are no studies quantifying the tissue levels of mucin subtypes in the colon of patients with DC or in individuals undergoing a therapeutic strategy that used a combination of both drugs for the treatment of the disease. Nevertheless, both SCF and NAC are easily available, low-cost substances, and their use in humans has been authorized by the main health agencies worldwide. Both substances are safe for use in humans with a small range of side effects.

## Conclusions

In the DC model used in the present study, the combination of SCF and NAC reduced the inflammatory process and increased the levels of the different mucin glycidic fractions present in the glands of colonic segments excluded from intestinal transit.

## Data Availability

The data will be available upon request.
